# Innate Allorecognition and Memory in Transplantation

**DOI:** 10.3389/fimmu.2020.00918

**Published:** 2020-05-28

**Authors:** Daqiang Zhao, Khodor I. Abou-Daya, Hehua Dai, Martin H. Oberbarnscheidt, Xian C. Li, Fadi G. Lakkis

**Affiliations:** ^1^Thomas E. Starzl Transplantation Institute, Department of Surgery, University of Pittsburgh, Pittsburgh, PA, United States; ^2^Department of Urology, Union Hospital, Tongji Medical College, Huazhong University of Science and Technology, Wuhan, China; ^3^Department of Immunology, University of Pittsburgh, Pittsburgh, PA, United States; ^4^Department of Critical Care Medicine, Center for Critical Care Nephrology, University of Pittsburgh, Pittsburgh, PA, United States; ^5^Immunobiology & Transplant Science Center and Department of Surgery, Houston Methodist Hospital, Texas Medical Center, Houston, TX, United States; ^6^Department of Medicine, Renal-Electrolyte Division, University of Pittsburgh, Pittsburgh, PA, United States

**Keywords:** allorecognition, innate immunity, transplantation, monocyte, dendritic cell

## Abstract

Over the past few decades, we have witnessed a decline in the rates of acute rejection without significant improvement in chronic rejection. Current treatment strategies principally target the adaptive immune response and not the innate response. Therefore, better understanding of innate immunity in transplantation and how to target it is highly desirable. Here, we review the latest advances in innate immunity in transplantation focusing on the roles and mechanisms of innate allorecognition and memory in myeloid cells. These novel concepts could explain why alloimmune response do not abate over time and shed light on new molecular pathways that can be interrupted to prevent or treat chronic rejection.

Activation of the innate immune system is necessary for driving adaptive immune responses (1, 2). In infection, pathogen-associated, non-self, molecules trigger host innate defenses and induce maturation of antigen-presenting cells (APCs) by binding to germline-encoded pattern recognition receptors [e.g., Toll-like receptors (TLRs)]. Mature APCs then initiate and sustain adaptive immunity by presenting antigen and providing co-stimulation to T cells.

How transplanted organs (allografts) induce APC maturation is less clear. Initial, landmark experiments suggested a role for TLRs by demonstrating that deletion of Myd88 downstream of TLRs blocks dendritic cell (DC) maturation and prevents rejection of single minor histocompatibility antigen-mismatched grafts (3). Later studies however showed that the rejection of MHC- or multiple minor antigen-mismatched allografts can still proceed in the absence of TLR signaling (4, 5). Moreover, deletion of additional microbial sensing pathways failed to completely prevent rejection (6–8). Similarly, the alternate hypothesis that “danger” molecules released at time of transplantation due to tissue injury trigger APC maturation could not account for alloimmune responses initiated after injury has subsided (9, 10). For example, allografts parked for a long time in T cell-deficient hosts were promptly rejected when T cells were replenished despite absence of discernible inflammation or injury in the graft at the time of T cell transfer (11–15). These observations raise the possibility that innate receptor systems, other than those involved in sensing microbes and danger, sense allogeneic non-self on transplanted tissues and cause APC activation. Here, we will summarize evidence that monocytes and macrophages distinguish between self and allogeneic non-self and review the mechanisms and functional consequences of this form of innate allorecognition. We also touch on the allospecific memory in these innate immune cells and discuss the translation of the findings into clinical situations.

## Evidence for Innate Allorecognition

An early study by Zecher et al. demonstrated that *RAG-/-* mice, which lack T and B cells, mount a DTH-like response to allogeneic but not syngeneic *RAG-/-* splenocytes (16). In the same study, it was established that the response is mediated by host monocytes, not NK cells, and is elicited by non-MHC disparities between donor and recipient. A subsequent publication by Liu et al. independently reported that macrophages in alloimmunized hosts engage in allorecognition, acquiring with the help of CD4+ T cells the ability to kill allogeneic cells (17). CD4+ T cell help to macrophages was mediated by CD40 such that the same macrophage allocytotoxic response could be elicited in lymphocyte-deficient mice injected with an anti-CD40 agonistic antibody at the time of alloimmunization.

Prompted by these observations, Oberbarnscheidt et al. studied the innate response of *RAG-/-*γ*c-/-* mice (which lack T, B, NK, as well as all other innate lymphoid cells) to heart, kidney, and bone marrow plug grafts (18). They found that allografts elicit an innate response distinct from syngeneic grafts. Allografts were persistently infiltrated with host-derived mature (MHC-IIhiCD80hi), IL-12+ monocyte-derived DCs (mo-DCs), even several weeks after transplantation, while syngeneic grafts harbored five-fold less mo-DCs, which were transient (present only during the 1st week), less mature, and IL-12neg. Similar differences were observed between allogeneic and syngeneic grafts transplanted to wild-type (WT) recipients and analyzed within 1 day after transplantation (18). Consistent with their IL-12 phenotype, mo-DCs from allografts but not those from syngeneic grafts drove a canonical Th1 (IFNγ+) response *in vitro* and *in vivo*. As in the previous study (16), the innate alloresponse was not dependent on MHC disparities between donor and recipient, or on lymphoid cells in either donor or recipient. Instead, a mismatch in the non-MHC was necessary. Chow et al. made similar observations by injecting allogeneic cells intravenously, to avoid inflammatory reactions, into *RAG-/-*γ*c-/-* mice (19). Therefore, monocytes and macrophages are activated by allogeneic stimuli to become mature DCs that drive the Th1 response and to acquire allocytotoxic functions, respectively.

## Mechanism of Innate Allorecognition: Recognition of Non-MHC Allodeterminants

**Figure 1:**
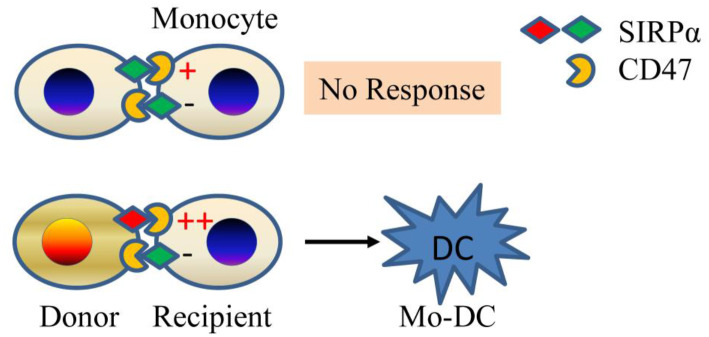
Innate allorecognition model. SIRPα mismatch between donor and recipient (bottom panel) causes imbalance between stimulatory and inhibitory signals in monocytes due to differential affinity of SIRPα to CD47. The mismatch generates mature DCs. If monocytes encounter self (top panel), then no response ensues. Mo-DC, monocyte-derived dendritic cell.

A genetic mapping study was undertaken to identify non-MHC allodeterminants that trigger the innate alloresponse (20). The study was based on the observation that allografts from NOD donors elicit a strong monocyte response in B6. *RAG-/-*γ*c-/-* recipients, while grafts from NOR mice, which share ~88% of their genome (including the MHC) with NOD, do not (20). Using NOD.NOR congenics, Dai et al. mapped the difference to the gene that encodes SIRPα (signal regulatory protein alpha), a polymorphic IgSF (immunoglobulin super family) protein expressed on neurons and myeloid cells but also present or induced on myocytes, epithelial cells, and endothelial cells (21). They showed that SIRPα triggers monocyte activation via CD47 and that amino acid polymorphisms in SIRPα determine the strength of the innate alloresponse by modulating binding to CD47 (20). The greater binding to its ligand CD47 by NOD variant of SIRPα than other mouse strains of SIRPα was also studied by other groups (22, 23). The allorecognition model (Figure 1) that emerged is that non-self SIRPα on donor cells causes host monocyte activation by disturbing the balance between activating and inhibitory signals mediated by CD47 and SIRPα, respectively. Under steady-state conditions, or upon transplanting a syngeneic graft, bidirectional interactions between CD47 and self-SIRPα are of equal affinity and thus prevent monocyte activation. In contrast, transplanting an allograft expressing a mismatched (non-self) SIRPα variant upsets the balance and causes monocyte differentiation to DC (20, 24). This model echoes NK cell allorecognition (25). At the same time, it does not exclude the possibility that other polymorphic ligands/receptors could still participate in fine-tuning the innate alloresponse.

## Allospecific Memory in Innate Immune Cells: Recognition of MHC-I Molecules

Immunological memory—the ability of immune cells to respond rapidly and provide enhanced protection of the host against previously encountered antigen—is a critical driver of transplant rejection and outcomes (26–28). Although originally confined to T & B lymphocytes, the memory concept has been expanded by discoveries that innate lymphoid and myeloid cells (NK cells and macrophages) (29–35), DCs (36), as well as non-immune cells (epithelial stem cells) (37) acquire memory to prior microbial, phagocytosis of apoptotic cells, or allogeneic exposures. As shown in Table 1, immunological memory is not a one-size-fits-all phenomenon but falls on a spectrum of varying biological mechanisms, ranging from epigenetic reprogramming in epithelial stem cells, macrophages, and DCs to clonal expansion and differentiation (with or without gene rearrangement) in NK cells and lymphocytes (36–42). Irrespective of mechanism, all memory enhances protection of the host. Epithelial stem cell memory hastens wound healing, macrophage or DC memory protects against pathogens, and lymphoid cell memory accelerates rejection of microbial and allogeneic non-self (31, 33–37, 43, 44). The lasting state of enhanced innate immunity, innate memory, had been termed “trained immunity” and usually confined to unspecific immunological memory in innate immune cells or does not have to be specific (45–49). Recent studies also revealed extensive changes in cellular metabolism during trained macrophage immunity, such as a switch from oxidative phosphorylation toward the preferential use of aerobic glycolysis through an Akt/mTOR/HIF-1α-dependent pathway induced by *C. albicans* and β-glutan (47). Strategies to regulate trained immunity had shown promise to achieve therapeutic benefits in a range of immune-related diseases (50).

**Table 1:** Spectrum of immunological memory.

	**Longevity**	**Recall**	**Specificity**	**Mechanisms**	**Enhanced protections**
Lymphocyte Memory	++++ (years)	++++	++++	Clonal Expansion Cell Differentiation Gene Rearrangement Epigenetic reprogramming	PATHOGENS: Yes ALLOANTIGENS: Yes
NK Cell Memory	++ (months)	++	++	Clonal expansion Cell differentiation Epigenetic reprogramming	PATHOGENS: Yes ALLOANTIGENS: Yes
Monocyte memory	+ (weeks)	+	++	Clonal expansion Cell differentiation Epigenetic reprogramming	PATHOGENS: Yes ALLOANTIGENS: Yes
Macrophage memory	+ (weeks)	+	+/-	Epigenetic reprogramming	PATHOGENS: Yes ALLOANTIGENS: Yes
DC memory	+ (weeks)	+	+	Epigenetic reprogramming	PATHOGENS: Yes ALLOANTIGENS: Yes
Epithelial stem cell memory	++ (months)	+	-	Epigenetic reprogramming	ENHANCED WOUND HEALING

In a series of experiments recently completed by our groups (51), we established that monocytes and macrophages mount an anamnestic memory response to previously encountered allogeneic donor cells but not to third-party cells. This donor specific feature was different from previous concept of “trained immunity,” suggesting it is similar to the well-characterized concept of antigen-specific immunological memory in adaptive immune cells (26–28). Memory arose independently of lymphoid cells in either the donor or recipient, underscoring its innate nature. It lasted between 4 and 7 weeks after immunization, which is significantly longer than the average lifespan of a monocyte (~3 days) (52, 53). Further, we established that memory specificity was to donor MHC-I antigens that were recognized by paired immunoglobulin-like receptor A (PIR-A) molecules expressed on monocytes and macrophages. PIR-A-/- mice or mice treated with PIR-A-blocking agents failed to mount monocyte or macrophage memory. Mouse PIRs are IgSF orthologs of human leukocyte immunoglobulin-like receptors (LILRs) (54). Six linked PIR-A and one PIR-B gene have been identified (55–57). PIR-B contains an ITIM motif and is inhibitory. It binds a wide spectrum of MHC-I molecules (58). PIR-As do not contain ITIM motifs and are stimulatory through association with the Fc receptor common γ (FcRγc) chain, also required for their surface expression (54, 59). PIR-A and PIR-B ectodomains share >92% identity, suggesting that PIR-As also bind MHC-I (58). In fact, PIR-A diversity leads to differential binding of individual PIR-A molecules to distinct MHC-I molecules.

**Figure 2:**
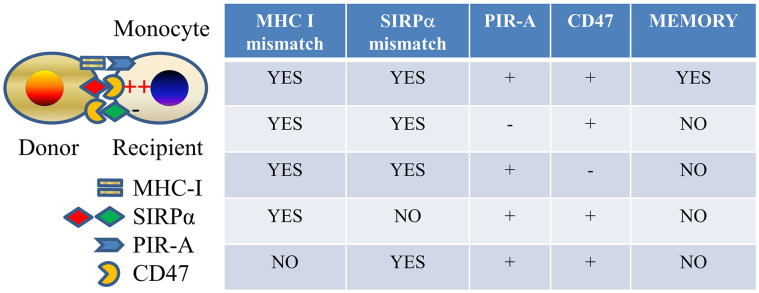
Allospecific innate memory mechanism. Mismatches of both MHC I and SIRPα between donor and recipient and expressions of both PIR-A and CD47 molecules on recipient monocytes are required for establishing monocyte allospecific memory.

As to the mechanisms by which monocytes acquire allospecific memory, the PIR molecules were preferentially expressed on Ly6Chi monocytes, which significantly expanded after allogeneic antigen exposure. Specific memory independent of lymphoid cells can be transferred to an unimmunized recipient by transferring sorted Ly6Chi monocytes expanded from an immunized recipient, suggesting that clonal expansion of monocytes that express the particular PIR-A molecule that recognizes the particular MHC-I molecule in the immunogen underlies memory (51). This resembles the mechanism established in the case of allospecific NK cell memory (34). We also observed that initial activation of monocytes via the SIRPα-CD47 pathway, which plays an important role in the primary innate allorecognition response (20), is necessary for priming cells toward the memory path (51) (Figure 2).

## Role of Innate Allorecognition in Rejection

Evidence that innate allorecognition described above plays an important role in rejection derives from three lines of investigation. In the first (18), OVA-specific OT-II T cells transferred to B6. *RAG-/-* hosts did not reject B6.OVA grafts but rejected (BALB/c x B6)F1.OVA grafts despite similar expression of the antigen, ovalbumin (OVA), recognized by the T cells. Only F1.OVA grafts induced mature mo-DCs and significant proliferation and IFNγ production by OT-II cells, underscoring the importance of monocyte recognition of allogeneic non-self in F1 donors in driving the T cell response. Moreover, short-term mo-DC depletion using the CD11b-DTR transgenic system completely abrogated histological acute rejection at 7 days in lymphocyte-replete mice (18). In contrast, eliminating neutrophils (also CD11b+) with a neutrophil-specific mAb did not affect rejection (18).

In the second study (60), the origin and function of DCs in heart and kidney allografts after transplantation to WT recipients were investigated. It was established that donor-derived DCs were quickly replaced by DCs derived from recipient monocytes and that they closely resembled mo-DCs generated by innate allorecognition. They were mature, IL-12+, and induced Th1 differentiation. In the graft, they made stable, cognate interactions with effector T cells and increased T cell proliferation and survival. DC depletion starting on day 5 delayed heart allograft rejection by >30 days in WT recipients (60) and completely prevented rejection in mice that lacked 2° lymphoid organs (splenectomized LTβR*-/-* mice) after transfer of effector T cells. Therefore, host mo-DCs that persistently infiltrate allografts sustain T cell-mediated rejection locally.

In the third set of experiments (51, 61), innate allorecognition and memory molecular pathways were interrupted. We observed that mouse renal allografts transplanted to recipients that lack either CD47 or PIR-A develop significantly less manifestations of chronic rejection. Similarly, blocking the PIR-A pathway led to long-term heart allograft survival with minimal pathology in recipients simultaneously treated with co-stimulation blockade (CTL4Ig). Acute rejection, however, was either not delayed or only modestly improved if either CD47 or PIR-A was absent. Therefore, the major influence of the SIRPα-CD47 and MHC-I-PIR-A pathways is on chronic allograft rejection and on preventing allograft acceptance. In contrast, rejection was accelerated in the absence of PIR-B signaling in the recipient.

## Clinical Translation in Transplantation

In humans, interactions through similar signaling pathways mediated by SIRPα and PIRs' homolog LILRs engaging with CD47 and MHC-I molecules, respectively, also exist (62, 63). By x-ray crystallography, Hatherley D et al. showed that the polymorphism in human SIRPα did not affect binding to its ligand CD47 (64). This suggested the possibility, although requiring further exploration, that human SIRPα differed in binding features from mouse SIRPα, whose binding affinity to its ligand CD47 was recognized to be dependent on its polymorphic IgV domain (20, 24). Our preliminary data also validated that the amino-terminal ligand binding domain of human SIRPα is highly polymorphic (65). Human LILRs family comprises a set of PIRs (A and B) expressed on myeloid innate immune cells. Similar to PIRs in mice, LILR-Bs contain ITIM motifs and are inhibitory while LILR-As do not contain ITIM motifs but contain ITAM motifs and are stimulatory. Six LILR-As and five LILR-Bs have been identified. Both LILR-As and LILR-Bs bind a wide spectrum of MHC-I molecules (63, 66). Human SIRPα-CD47 interaction has been reported to be implicated in the phagocytosis of red blood cells and leukemia cells by macrophages *in vivo* or *in vitro* (62, 67). There are data suggesting a link between LILR polymorphism and control of HIV infection and autoimmunity in humans (63, 66). However, published human studies on the roles of SIRPα and LILRs in transplantation are not available yet. The similarities in these two pathways (SIRPα-CD47 and MHC-I-PIR-A/LILR-A) between human and mice should trigger investigations into the roles of these pathways in clinical transplantation.

## Concluding Remarks

We have presented evidence that the innate immune cells, namely, monocytes and macrophages, respond to allogeneic non-self independently of T, B, and NK cells. This form of allorecognition initiates or sustains the responses of recipient T cells to allografts by inducing the maturation of APCs. It also provides phagocytic cells with the means to kill allogeneic targets without inflicting damage on self-tissues. One mechanism of innate allorecognition is the differential binding of CD47 on monocytes to polymorphic SIRPα on donor cells. We also summarized data showing that monocytes and macrophages acquire memory specific to allogeneic MHC-I molecules that is dependent on MHC-I sensing by polymorphic PIR-A molecules. Blocking SIRPα-CD47 or MHC-I-PIR-A interaction shows promise in preventing chronic rejection or promoting allograft acceptance. Future studies are expected to establish translation of these findings into clinical transplantation.

## Author Contributions

DZ and FL wrote manuscript. KA-D and HD contributed data and edited manuscript. MO and XL edited manuscript.

## Conflict of Interest

The authors declare that the research was conducted in the absence of any commercial or financial relationships that could be construed as a potential conflict of interest.
